# A Combination of Immune Checkpoint Inhibition with Metronomic Chemotherapy as a Way of Targeting Therapy-Resistant Cancer Cells

**DOI:** 10.3390/ijms18102134

**Published:** 2017-10-13

**Authors:** Irina Kareva

**Affiliations:** 1 Mathematical and Computational Sciences Center, School of Human Evolution and Social Change, Arizona State University, Tempe, AZ 85287, USA; ikareva@asu.edu; Tel.: +1-(978)-294-1100; 2 EMD Serono Research and Development Institute, Merck KGaA, Billerica, MA 02370, USA

**Keywords:** immune checkpoint inhibitors, combination therapy, metronomic chemotherapy, tumor microenvironment, metabolic competition, MTD

## Abstract

Therapeutic resistance remains a major obstacle in treating many cancers, particularly in advanced stages. It is likely that cytotoxic lymphocytes (CTLs) have the potential to eliminate therapy-resistant cancer cells. However, their effectiveness may be limited either by the immunosuppressive tumor microenvironment, or by immune cell death induced by cytotoxic treatments. High-frequency low-dose (also known as metronomic) chemotherapy can help improve the activity of CTLs by providing sufficient stimulation for cytotoxic immune cells without excessive depletion. Additionally, therapy-induced removal of tumor cells that compete for shared nutrients may also facilitate tumor infiltration by CTLs, further improving prognosis. Metronomic chemotherapy can also decrease the number of immunosuppressive cells in the tumor microenvironment, including regulatory T cells (Tregs) and myeloid-derived suppressor cells (MDSCs). Immune checkpoint inhibition can further augment anti-tumor immune responses by maintaining T cells in an activated state. Combining immune checkpoint inhibition with metronomic administration of chemotherapeutic drugs may create a synergistic effect that augments anti-tumor immune responses and clears metabolic competition. This would allow immune-mediated elimination of therapy-resistant cancer cells, an effect that may be unattainable by using either therapeutic modality alone.

## 1. Introduction

The initial significant successes in curative chemotherapy occurred in the 1960s, when a combination of simultaneously administered chemotherapeutic drugs induced long-term remissions in children with acute lymphoblastic leukemia (ALL). A similar approach has proven successful for Hodgkin’s disease, testicular cancer, as well as in large part for non-Hodgkins lymphoma and some leukemias, which until then were largely fatal [1,2,3]. This was when the maximal tolerated dose (MTD) protocol was established, which involves administering the highest tolerable dose of the chemotherapeutic agent just short of unacceptable toxicity.

The MTD approach was developed prior to the realization driven by the rise of molecular biology in the 1970s, that most cancers are genetically heterogeneous, to which ALL is a rare exception. MTD-type treatment, which applies severe selective pressure to a heterogeneous cancer cell population, naturally results in removal of therapy-sensitive cell clones, leaving therapy-resistant clones to regrow between cycles. And while, in theory, administration of a high enough dose might remove all the cancer cells, in practice, such doses would be too high to be tolerated by the patient. For this reason, the drug is administered in cycles, with large intervals between treatments to allow the patient to partially recover.

Furthermore, in recent years it has become increasingly recognized that most tumors engage and modify their microenvironment. They create a niche for themselves, and circumvent the immune system [3,4,5,6,7,8,9,10,11], thus further reducing the efficacy of the standard therapeutic approach.

### Tumors Engage and Modify Their Microenvironment

One of the mechanisms whereby many solid tumors engage and modify their microenvironments is through upregulation of the anaerobic metabolism of glucose (glycolysis). This natural adaptation to diminishing oxygen supply results from increased cell proliferation and hence greater inter-cellular competition for shared resources in the tumor microenvironment. Increase in glycolysis has two important implications.

Firstly, the accumulation of lactic acid (a by-product of anaerobic metabolism) can create an immune-suppressive environment, promoting activation of naïve lymphocytes away from the tumor-suppressing Th1 phenotype and towards Th2 tumor-promoting phenotype [5]. This effectively decreases the number of cytotoxic lymphocytes that are capable of tumor elimination.

Secondly, upregulation of glycolysis by cancer cells is accompanied by 20–40-fold increase in the upregulation of nutrient transporters, since anaerobic metabolism yields ATPs (adenosine triphosphate, also referred to as “molecular currency unit” used for intracellular energy transfer) per glucose molecule compared to up to 30 ATPs per molecule of oxidized glucose [5,12,13]. The low energy yield of glycolytic mode of glucose metabolism naturally requires larger amounts of glucose to meet each cell’s basic needs. However, cytotoxic lymphocytes also require large amounts of glucose to enable both mobility and cytotoxic function; in fact, they lose cytotoxic functionality in the state of nutrient deprivation [14,15]. Therefore, in the tumor microenvironment there exists competition for shared nutrients between anaerobic cancer cells and cytotoxic lymphocytes. Failure of the immune cells to outcompete cancer cells for shared nutrients results in suppression of anti-tumor immune responses, a mechanism that was theoretically predicted by Kareva and Hahnfeldt [5], investigated mathematically by Kareva and Berezovskaya [16] and later experimentally confirmed in [6].

If chemotherapeutic treatment under the MTD regimen is administered to such a tumor, not only does it result in the aforementioned selection for therapy-resistant clones via removal of sensitive cell clones, but it also leaves behind a microenvironment that has additionally been primed to favor the remaining cancer cells [4,7]. Furthermore, since apoptotic and necrotic cells release their intracellular stores of nutrients, such as glucose and phosphorus, into their microenvironment, they provide additional sources of nutrients and building materials for the remaining therapy-resistant cancer cells [17,18,19,20], allowing them to more rapidly repopulate the tumor.

## 2. Alternative to Maximal Tolerated Dose (MTD): Metronomic Chemotherapy

**Figure 1:**
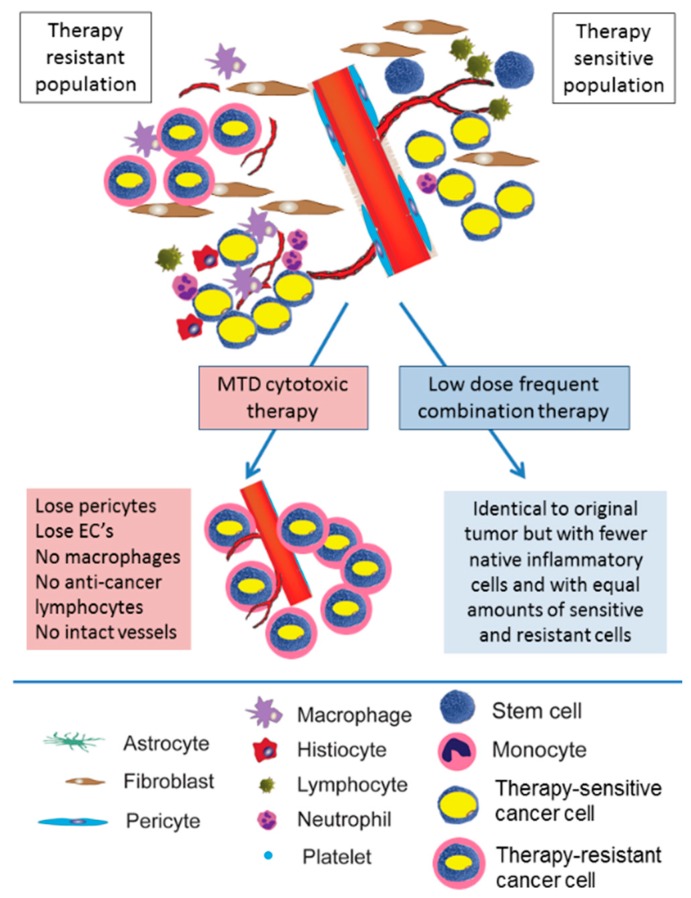
Comparison of the effects of maximal tolerated dose (MTD) and metronomic chemotherapy. MTD cytotoxic chemotherapy results in the ablation of anti-tumor immunity and the elimination of therapy-sensitive clones. In turn, these result in the selection of therapy-resistant cancer cells. In contrast, low-dose high-frequency (metronomic) chemotherapy targets the tumor stroma, gradually reducing tumor size but mostly maintaining tumor composition, decreasing therapeutic resistance and maintaining anti-tumor immunity. Figure is adapted from [26].

An alternative approach to chemotherapy administration involves more frequent administration of lower doses of cytotoxic agents, also referred to as metronomic therapy [21,22,23,24,25]. It provides numerous advantages compared to the standard MTD protocol, including a decrease in tumor vascularization, lower therapeutic resistance, and, perhaps most importantly, augmented anti-tumor immune responses [21]. All of these mechanisms will be described in greater detail below, and are summarized in Figure 1.

### 2.1. Decreased Angiogenesis

Tumors cannot grow beyond a size of 1–2 mm^3^ without securing their own blood supply, a process known as angiogenesis [27,28]. The process of both normal and pathological vascularization is mediated by the tightly regulated sequential release of pro- and anti- angiogenesis growth factors from platelets, the megakaryocyte-derived circulating microparticles involved in wound healing [29]. In the event of a wound, first pro-angiogenesis regulators, such as VEGF, are released from platelets, initiating formation of vessel tips [30,31]. Next, growth factors responsible for vessel stalk formation are released. These factors include bFGF and PDGF [31,32]. Finally, angiogenesis inhibitors are released from the platelets, causing apoptosis of proliferating cells and consequently vessel pruning and termination of angiogenesis [33,34,35,36]. Angiogenesis inhibitors include angiostatin, endostatin, PF-4, among others. In order to signal, all angiogenesis regulators must bind to glycosaminoglycans (GAGs), such as heparan sulfate (HS), on the cell surface [37,38]. In tumors, cancer cells induce the surrounding stroma to produce additional stimulators of angiogenesis, which may outcompete angiogenesis inhibitors for HS much like in a game of musical chairs. That prevents the angiogenesis inhibitors from signaling and thus precludes the termination of blood vessel formation [39].

Low-dose high-frequency chemotherapy aims to target not the cancer cells themselves but the supporting stroma, which is the source of angiogenesis regulators. Removing the source of angiogenesis stimulators gives angiogenesis inhibitors an opportunity to signal and terminate the process of blood vessel formation.

### 2.2. Decrease Therapeutic Resistance

Cancer cells depend on supporting stroma to provide pro-angiogenic signaling that would allow the formation of blood vessels needed for recruiting nutrients and oxygen [27,39,40]. However, stromal cells, such as fibroblasts and pericytes, can be more sensitive to chemotherapeutic agents and can also be damaged by doses that are not harmful to cancer cells [3]. Therefore, targeting tumor stroma would inflict equal damage on the supply compartment that supports both sensitive and resistant cells [21]. This can weaken the entire tumor population without selecting for resistant clones, particularly in combination with other treatment modalities [24,41,42,43,44,45].

Notably, a common argument against low-dose administration of chemotherapeutic agents stems from comparison with the low efficacy of antibiotics in fighting bacterial infections [46]. The critical difference lies in the fact that antibiotics, at any dose, target the bacteria directly, while low-dose chemotherapy is aimed to target not the cancer cells but the supporting stroma. Therefore, mechanisms responsible for emergence of antibiotic resistance are largely inapplicable to low-dose chemotherapy administration.

### 2.3. Promote Anti-Tumor Immunity

The functionality of the immune system can be compromised by high-dose chemotherapy as immune cells can be ablated by cytotoxic drugs and thus prevented from pursuing therapy-resistant cancer cells. However, metronomically administered chemotherapy can increase the ablation of immunosuppressive regulatory T cells (Tregs) [47,48,49], promote the maturation of antigen presenting cells [50], improve the activity of dendritic cells (DCs) [51] and, most importantly, improve the activation and functionality of cytotoxic NK and CD8^+^ T cells [45,52,53].

Specifically, Doloff and Waxman [54] demonstrated that administration of metronomic cyclophosphamide every six days (Q6day cycle) resulted in significant recruitment and activation of natural killer (NK) cells, dendritic cells and macrophages. This response was accompanied by a dramatic regression of implanted glioma xenografts. The mechanism is contingent on the concurrent active signaling of the VEGFR2 receptor [54]. More frequent administration of cyclophosphamide inflicted severe damage on the NK cells themselves, while less frequent Q9day and Q12day schedules eventually resulted in tumor escape. Similar results were obtained for increasing activation and functionality of CD8^+^ T cells [52]. The authors’ results demonstrate that finding an appropriate timing and dosing schedule might dramatically improve treatment outcome by both engaging and protecting anti-tumor immune responses. Finding such a combination for each cancer type still remains a challenge to be addressed in future research.

Furthermore, metronomic chemotherapy may provide a way to target other immunosuppressive components of the tumor microenvironment, such as myeloid derived suppressor cells (MDSCs). MDSCs are a heterogeneous group of immune cells of myeloid lineage characterized by their immature state and ability to suppress T cells [55]. For instance, Highfill et al. [56] demonstrated that the accumulation of myeloid-derived suppressor cells in the tumor bed can limit the efficacy of checkpoint blockade in cancer. MDSCs, however, can be effectively targeted by chemotherapeutic agents, such as docetaxel, clusterin [57] and gemcitabine [58], as well as a combination of chemoimmunotherapies [59], alleviating immune cell suppression and promoting anti-tumor responses.

### 2.4. Targeting Cancer Stem Cells

Metronomic chemotherapy can additionally provide a way of targeting cancer stem cells (CSCs) and stem-like tumor-initiating cells (TICs), which cannot be achieved with a standard MTD approach. For instance, Chan et al. [60,61] showed that MTD-induced activity in carcinoma-associated fibroblasts resulted in signaling that triggered phenotypic conversion of carcinoma cells into stem-like tumor-initiating cells (TICs), which led to increased invasive behavior. They investigated this via the molecular analysis of tumor stroma in both neoadjuvant chemotherapy-treated human desmoplastic cancers and in orthotropic tumor xenografts. In contrast, the same overall dose administered on a metronomic schedule largely prevented therapy-induced phenotypic conversion into TICs, enhancing treatment response and extending survival of tumor-bearing mice. This study highlighted the crucial contribution of stroma in cancer treatment and a need to be cognizant of the systemic nature of the disease in devising treatment approaches.

Furthermore, Relation et al. [62] reviewed challenges associated with targeting cancer stem cells (CSCs), which might be overcome by changing approaches to therapy administration. Specifically, the authors highlighted CSC-specific differences that make them particularly difficult to target therapeutically. For instance, traditional chemotherapeutic treatments target primarily rapidly-dividing cells, thus allowing slower-dividing CSCs to evade destruction [63]. Furthermore, CSCs can upregulate checkpoint regulators, such as Rad17 and Chk1/2 after treatment [64], decreasing their sensitivity to the immune system [65]. Augmenting immune response as a way to target CSCs might provide improved therapeutic outcomes. That might be achieved by using metronomic chemotherapy to increase immune cell access to CSCs in the tumor, possibly coupled with immune checkpoint inhibition.

## 3. Checkpoint Inhibitors

It is likely that engaging the body’s anti-tumor immunity might hold the key to targeting therapy-resistant cells, provided that cytotoxic cells are not ablated by the treatment, and are capable of tumor infiltration. A possible way to achieve this effect can be to combine metronomically-administered chemotherapy with immunotherapy, and in particular, with one of the most promising types of immunotherapy, namely, immune checkpoint inhibition.

Immune checkpoint inhibitors target mechanisms that regulate immune cell activation and cytotoxic function against self-antigens as a protection against auto-immune disease. Alleviating some degree of checkpoint activity has been shown to significantly augment immune responses [66], leading to improved outcomes in cancer patients [67]. The two currently most studied targets involve inhibition either of the cytotoxic T-lymphocyte antigen-4 (CTLA-4) cell surface receptor, the programmed cell death-1 (PD-1) surface receptor, or the corresponding soluble PD-L1 or PD-L2 ligands [68].

### 3.1. CTLA-4 Inhibition

**Figure 2:**
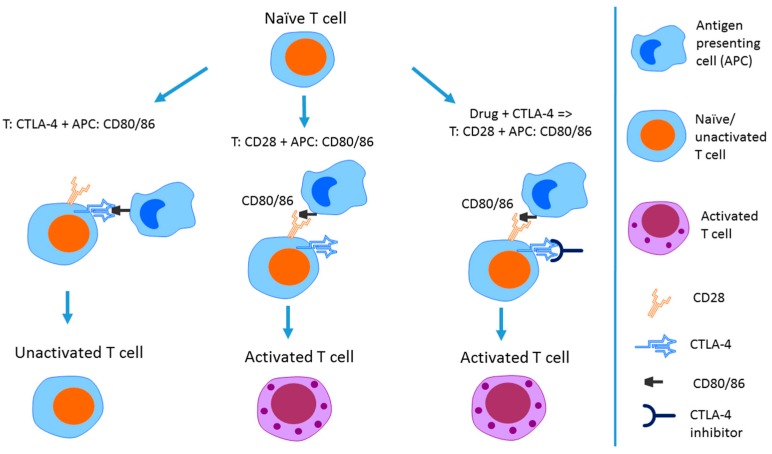
Mechanism of action of CTLA-4 checkpoint inhibitor. CTLA-4 and CD28 are receptors expressed on the T cell surface; CD80/86 are receptors on the surface of antigen-presenting cells (APCs). When CTLA-4 comes in contact with CD80/86 receptors, the T cell remains unactivated. Interaction of CD28 on T cell surface with CD80/86 on the APC cell surface results in T cell activation. Pharmacological blocking of CTLA-4 on the T cell surface increases the likelihood of CD28–CD80/86 binding, resulting in maintenance of T cell activation. Figure is adapted with permission from [26].

Activation of naïve T cells is mediated by interactions with antigen-presenting cells (APCs), such as dendritic cells, which result in the formation of specialized structures at points of APC and T cell contact (immunological synapses) [69]. T cells express proteins on the cell surface that provide co-stimulatory signals to activate or suppress T cell activation and survival, including cluster of differentiation 28 (CD28) and CTLA-4. Binding of CD80 or CD86 receptors on the APC surface to CTLA-4 on T cells results in blocking T cell activation. Alternatively, binding of CD80/86 to T-cell receptor CD28 results in increasing T cell activation [68,70,71]. Pharmacological blockade of CTLA-4 gives a competitive advantage to CD28, resulting in increased T cell activity. This mechanism is summarized in Figure 2.

A CTLA-4 immune checkpoint inhibitor interferes with potentially autoreactive T cells in the earlier stages of T cell activation, primarily in lymph nodes [71,72]. The first FDA-approved checkpoint inhibitor, which acts by blocking the CTLA-4 receptor, is ipilimumab, a monoclonal antibody approved in 2011 for treatment of unresectable or metastatic melanoma [73]. It has shown much promise, and is currently undergoing clinical trials for treatment of other cancers, including lung, kidney and prostate cancers (Phase III), as well as cervical, colorectal, gastric, pancreatic, ovarian and urothelial cancers (Phase II).

### 3.2. PD-1, PD-L1 and PD-L2 Inhibition

Programmed cell death protein 1 (PD-1) is a cell surface receptor that regulates T cell activation through binding to soluble ligands PD-L1 and PD-L2 [68,74]. Similarly to CTLA-4, PD-1 signaling interferes with T cell proliferation, glucose metabolism and cytokine signaling. It promotes apoptosis in antigen-specific T cells, and reduces apoptosis of Tregs, indirectly increasing immune cell regulation [68,75,76,77]. Increased expression of PD-1 is one of the hallmarks of T cell exhaustion [78]. Some important differences between the two mechanisms lie in the fact that while CTLA-4 regulates T cell activation early in the immune response, primarily in the lymph nodes, PD-1 affects T cell effector responses in the peripheral tissues [72]. Furthermore, while CTLA-4 expression is restricted to T cells, PD-1 is expressed by T cells, B cells and macrophages; it can also be expressed on other cells, including some tumor cells [74,79,80].

The PD-L1 and PD-L2 ligands are expressed on leukocytes, macrophages and dendritic cells; PD-L1 has also been found on many tumor types and has been associated with poor prognosis [80]. Since PD-1 ligands are expressed in peripheral tissues, these complexes are believed to maintain self-tolerance in locally infiltrated tissues [72]. Pharmacological interventions for augmenting immune cell responses involve either blocking the PD-1 cell surface receptor, or blocking the soluble ligands PD-L1 and PD-L2 in order to interfere with complex formation. Currently approved anti-PD-1 drugs include pembrolizumab, which is used for treating unresectable or metastatic melanoma, and metastatic non-small cell lung cancer. Also included is nivolumab, which is used for the second and third line treatment of unresectable or metastatic melanoma, and for the treatment of metastatic non-small cell lung cancer, advanced renal cell carcinoma (RCC), relapsed or progressed Hodgkin lymphoma, recurrent or metastatic squamous cell carcinoma of head and heck, advanced or metastatic urothelial carcinoma, colorectal cancer and hepatocellular carcinoma. Anti-PD-L1 drugs durvalumab and atezolizumab have entered phase III clinical trials for head and neck cancer (durvalumab), bladder cancer (atezolizumab) and lung cancer (both), and Phase II trials for colorectal cancer and glioblastoma (durvalumab) and kidney cancer (atezolizumab). Durvalumab has been approved for the treatment of advanced or metastatic urothelial carcinoma. Atezolizumab has been approved for the treatment of locally advanced or metastatic urothelial carcinoma and metastatic non-small cell lung cancer. The anti-PD-L1 checkpoint inhibitor avelumab was recently approved for the treatment of metastatic Merkel cell carcinoma (MCC) and urothelial cancer.

Immune checkpoint inhibition has been gaining success, particularly in metastatic cancers, which still remain the cause of the majority of cancer-related deaths. It is possible that the effect can be further augmented by combining it with metronomically-administered chemotherapy due to synergistic mechanisms that are outlined in the following section.

## 4. Combination: Metronomic Chemotherapy and Checkpoint Inhibitors

Based on the mechanisms of action of both metronomic chemotherapy and immune checkpoint inhibitors, one can surmise that a combination of the two therapeutic approaches would have a synergistic effect for the following reasons:

(1) Both immune checkpoint inhibitors and metronomic chemotherapy increase immune cell activation. While metronomic chemotherapy can promote tumor-specific immune activation, concurrent administration of immune checkpoint inhibitors would maintain the activated state of T cells.
(2) Administration of metronomic chemotherapy would allow competition for nutrients between tumor and immune cells to be reduced via gradual removal of tumor cells. This would facilitate tumor infiltration by cytotoxic immune cells, which has been associated with improved clinical outcome.
(3) Experimental evidence [6] has shown that blocking PD-L1 directly on tumors dampens glycolysis, giving cytotoxic lymphocytes additional competitive advantage.

**Figure 3:**
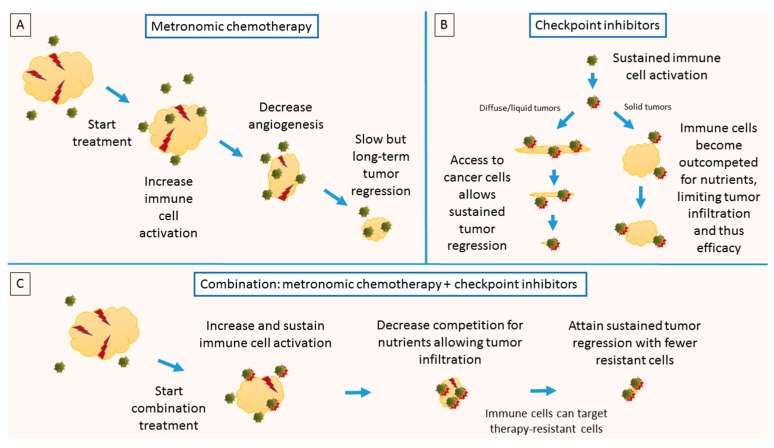
Synergistic effects of metronomic chemotherapy combined with checkpoint inhibition. (**A**) Metronomic chemotherapy causes decrease in angiogenesis and maintained anti-tumor immunity, resulting in slow but long-term tumor regression; (**B**) checkpoint inhibition results in sustained immune cell activation; the therapeutic effect is particularly strong in liquid or diffused tumors but is limited in solid tumors due to low tumor infiltration; (**C**) combination of the two therapeutic modalities would create a synergistic effect: metronomic chemotherapy would increase immune activation and facilitate tumor infiltration by removing metabolic competition between tumor and immune cells. Checkpoint inhibition would maintain immune activation, allowing effective elimination of therapy-resistant cells as the immune cells are now capable of infiltrating the tumor core. Figure is adapted with permission from [26].

These considerations have been summarized in Figure 3.

In summary, a combination of immune checkpoint inhibitors and metronomic chemotherapy may provide an avenue for targeting therapy-resistant cells, including CSCs and TICs, without inflicting unacceptable toxicity, resulting in high treatment compliance, improved long-term outcomes for difficult-to-treat cancers, and improved patient quality of life.
